# Clinical and Radiological Outcomes of Single-Level, Stand-Alone LLIF Without Supplemental Posterior Fixation: A Retrospective Cohort Study

**DOI:** 10.3390/medicina62071414

**Published:** 2026-07-21

**Authors:** Grzegorz Guzik, Dariusz Sowa, Dawid Merkiel, Michał Bronisz

**Affiliations:** 1 Department of Orthopaedic Oncology, Subcarpatian Oncology Centre, 36-200 Brzozow, Poland; 2 Trauma and Orthopaedic Department, District Hospital in Stalowa Wola, 37-450 Stalowa Wola, Poland; sowad@op.pl (D.S.); dawid220894@gmail.com (D.M.);

**Keywords:** spine surgery, interbody fusion, canal stenosis, spine spondylosis, lumbar discopathy

## Abstract

*Background and Objectives*: The aim of this study was to use a modified lateral lumbar approach developed by the authors for single-level lumbar decompression and interbody fusion using intervertebral implants. *Materials and Methods*: A total of 38 patients with single-level L1–L5 degenerative disc disease who underwent lumbar interbody fusion were included in this study. The procedure was performed using the modified lateral lumbar surgical approach. Clinical outcomes included the assessment of functional status using the Karnofsky Performance Scale and Oswestry Disability Index (ODI), quality of life using the EuroQol Visual Analogue Scale (EQ-VAS), and pain intensity using Visual Analogue Scale (VAS). Preoperative and postoperative radiographic parameters assessed on magnetic resonance imaging (MRI) were compared. *Results*: Significant improvement in functional status, quality of life, and pain reduction was observed in all patients. Radiographic outcomes also improved significantly. Mean disc height increased from 8.2 mm preoperatively to 12.2 mm postoperatively (*p* < 0.05). The cross-sectional area of the spinal canal increased significantly following surgery. Furthermore, a reduction in disc herniation width and enlargement of the intervertebral foramina were observed. No neurovascular complications occurred, and endplate injury with cage subsidence was noted in 5% of patients. *Conclusions*: Lateral lumbar discectomy and interbody fusion increase spinal canal area, enlarge the neuroforamina, and restore intervertebral disc height. Most patients experienced substantial functional improvement and pain reduction. No neurological or vascular complications were observed, which may be due to the modified approach. The confirmation of this hypothesis requires further research and a longer follow-up period.

## 1. Introduction

Minimally invasive surgical techniques, such as lateral lumbar interbody fusion (LLIF), are increasingly used in the treatment of central and lateral lumbar spinal stenosis, degenerative scoliosis, spondylolisthesis, and spinal infections, including discitis [1,2].

Lateral interbody fusion enables indirect neural decompression, restoration of sagittal and coronal spinal alignment, reconstruction of intervertebral disc height, and achievement of interbody fusion. Compared with traditional posterior approaches, lateral spinal approaches reduce paraspinal muscle and soft tissue injury, facilitate wound healing, and are associated with reduced postoperative pain, a shorter operative time, a shorter hospital stay, and lower rates of postoperative wound infection. LLIF may be performed as a stand-alone procedure or combined with supplemental posterior transpedicular fixation [3,4,5,6,7].

Radiographic studies have demonstrated that the increase in the spinal canal cross-sectional area following LLIF may be smaller compared with direct posterior decompression techniques. Reported complications include implant migration, cage subsidence into the vertebral endplates, and vascular or neural injuries, particularly those involving the genitofemoral nerve. In addition, the LLIF approach may be technically challenging or infeasible at the L4–L5 level due to a high-riding iliac crest. The overall complication rate has been estimated at approximately 18%, while severe complications occur in less than 1% of cases. The reported reoperation rate is approximately 2%. Severe lumbar spinal stenosis may represent a relative contraindication to indirect decompression techniques [5,6,7,8,9,10,11].

**Figure 1:**
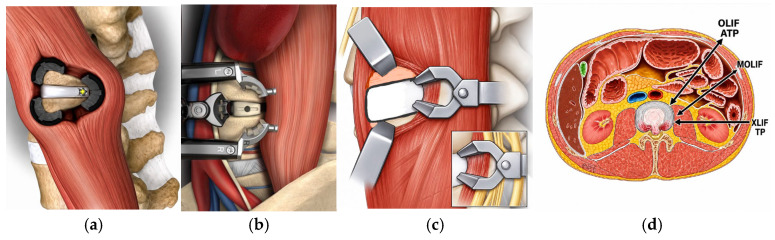
Two principal techniques for lateral lumbar interbody cage implantation. XLIF-TP (extreme lateral interbody fusion—transpsoas) is presented in (**a**), and OLIF-ATP (oblique lumbar interbody fusion—anterior to psoas) is presented in (**b**). The MOLIF (modified oblique lumbar interbody fusion) approach described in this study is presented in (**c**). The cross-section of the lumbar spine with a diagram of the surgical approaches is presented in (**d**).

The most commonly used lateral retroperitoneal approach provides access to the L1–L5 intervertebral spaces and allows for the placement of interbody cages. Two principal techniques of deep tissue dissection have been described (Figure 1). The first involves direct transpsoas cage insertion using dedicated retractor systems and instrumentation (XLIF-TP). Although this technique minimizes the risk of vascular injury, it is associated with a higher incidence of transient neurological complications. The second technique involves dissection along the anterior border of the iliopsoas muscle with posterior retraction of the muscle (OLIF- ATP). This approach may reduce the risk of neural injury; however, it carries a potentially higher risk of vascular complications [1,7,8,12].

The aim of this study was to describe and evaluate the functional, surgical, and radiographic outcomes of a modified lateral lumbar approach developed by the authors for single-level lumbar decompression and interbody fusion using intervertebral implants. We hypothesized that the use of stand-alone, single-level LLIF would provide good clinical and radiographic outcomes with a low risk of complications.

## 2. Material and Methods

We retrospectively reviewed the medical records of 38 patients who underwent single-level, stand-alone lateral interbody fusion (LLIF) between January and December 2021 at the Department of Orthopaedic Oncology in Brzozów and the Department of Trauma and Orthopaedic Surgery in Stalowa Wola.

The majority of patients were male (60%; 23 patients). The mean ages were 61 years in female patients and 65 years in male patients.

The inclusion criteria for this study were symptomatic single-level degenerative disc disease involving the L1–L5 lumbar spine segments. No additional criteria were applied, considering the morphology of the discopathy.

The exclusion criteria were the presence of multilevel disc disease, the coexistence of vertebral canal stenosis caused by hypertrophy of the ligament flavum and/or intervertebral joints, asymptomatic patients, osteoporosis, a history of vertebral body fractures, and transpedicular spine fixations.

The most frequently treated level was L4–L5 (16 patients), followed by L2–L3 (10 patients), L3–L4 (8 patients), and L1–L2 (4 patients). No supplemental posterior fixation was performed in any patient.

**Figure 2:**
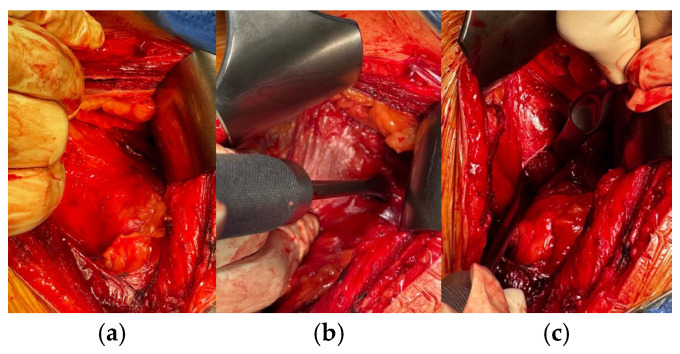
The modified approach used in this study. (**a**) An intraoperative image of the psoas muscle. (**b**) Separation under direct visualization at 1 cm anterior to the psoas muscle using a Cobb elevator. This protects the vascular structures from injury. The posterior part of the psoas muscle is then gently mobilized and retracted posteriorly off the disc, thereby protecting the lumbar plexus nerves from injury, as shown in (**c**).

The surgical procedure was performed using a modified lateral lumbar approach developed by the authors—MOLIF. In this surgical procedure, a 6 cm oblique skin incision was made on the right side of the abdomen. The abdominal wall muscles were separated along the direction of their fibres, followed by the incision of the transversalis fascia. The border of the iliopsoas muscle was identified visually, and the muscle fibres were longitudinally separated using a Cobb elevator approximately 1 cm posterior to its anterior border. Subsequently, the muscle was retracted posteriorly. After the lateral aspect of the intervertebral disc was adequately exposed, dedicated illuminated retractors were inserted and stabilized to the vertebral bodies using threaded fixation pins. This semi-open technique was intended to minimize the risk of vascular and neural injuries (Figure 2).

**Figure 3:**
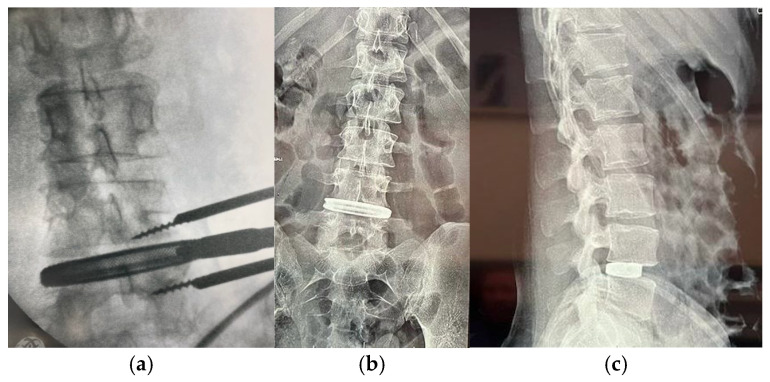
Stages of discectomy and cage implantation (**a**–**c**).

A window was then created in the annulus fibrosus, followed by discectomy and endplate preparation. Particular care was taken to preserve the vertebral endplates in order to reduce the risk of cage subsidence. Following cage implantation and the fluoroscopic verification of implant position, the wound was closed without drain placement (Figure 3).

Regarding neurological examination, functional status, assessed using the Karnofsky Performance Scale and Oswestry Disability Index (ODI); quality of life, assessed using the EuroQol Visual Analogue Scale (EQ-VAS); and pain intensity, assessed using a Visual Analogue Scale (VAS), were evaluated preoperatively, as well as 7 days and 3 months postoperatively [13,14,15,16].

Preoperative and postoperative magnetic resonance imaging (MRI) examinations were used to evaluate the following radiographic parameters: the cross-sectional area of the spinal canal, height and width of the intervertebral foramina, width and depth of disc extrusion, and width of the lateral recesses. Sagittal T2-weighted sequences were used to assess the size (height and depth) of the disc protrusion and the height and width of the intervertebral foramen. Assessments of the cross-sectional spinal canal area and the width of lateral recess were performed in axial T2-weighted sequences. The osseointegration of the implants was assessed in sagittal T2-weighted and STIR sequences. The parameters indicative of fusion were the absence of liquid and/or a gap between the bone and the implant, the absence of bone marrow edema in the adjacent vertebrae, and tight adherence of the implants to the bone. A 3 Tesla GE (General Electric) SIGNA device was used in this study. The measurements were performed in a typical manner. Computer tomography, which provides the best assessment of bone–implant fusion, was not performed because of concerns regarding patient exposure to ionizing radiation.

Standing anteroposterior and lateral radiographs were obtained one day before surgery, as well as one day and three months postoperatively. Anterior disc height, lumbar lordosis angle, and segmental lordosis angle were measured preoperatively and postoperatively. Implant position was carefully assessed with particular attention to implant migration and subsidence.

The radiographic and MRI parameters were independently evaluated by the operating orthopedic surgeon and a radiologist. No discrepancies were observed between the assessments made by the orthopedic surgeon and the radiologist; therefore, the results are presented as the means of the two evaluations.

General and surgical complications, including infection, vascular injury, neural injury, and intraoperative bleeding, were analyzed.

Statistical analyses examined pain intensity based on the VAS score, functional status based on the Karnofsky Performance Scale, functional disability based on the ODI questionnaire, and quality of life based on the EQ-VAS score. For continuous variables with a normal distribution, the results were presented as the mean ± standard deviation. Differences between preoperative and postoperative values were analyzed using a paired Student’s *t*-test. Scores on the Karnofsky Performance Scale presented a skewed distribution and were therefore subjected to logarithmic transformation to normalize the data distribution. These data were presented as median values, and variability was analyzed using the paired *t*-test for normalized variables. Categorical variables were presented as percentages, and intergroup differences were analyzed using the chi-square test. A *p*-value < 0.05 was considered statistically significant. Statistical analyses were performed using STATISTICA version 13.0 software (StatSoft Inc., Tulsa, OK, USA).

## 3. Results

Significant improvements in functional status, quality of life, and pain reduction were observed in all patients (Table 1).

Preoperative pain intensity assessed using the Visual Analogue Scale (VAS) ranged from 5 to 9 points, with a median value of 7. Seven days after surgery, the patients’ VAS scores ranged from 2 to 4, with a median value of 3. At 3-month follow-up, their VAS scores ranged from 1 to 2 points, with a median value of 1.5.

Preoperative functional status assessed using the Karnofsky Performance Scale ranged from 30 to 100, with a median score of 50. Seven days after surgery, the patients’ scores ranged from 30 to 100, with a median value of 70. Three months postoperatively, their scores ranged from 30 to 100, with a median value of 90 (*p* < 0.05).

The mean preoperative Oswestry Disability Index (ODI) score was 51.2. Seven days and 3 months after surgery, the mean ODI values decreased to 31.8 and 14.9, respectively (*p* < 0.05). The mean preoperative quality-of-life score according to the EQ-VAS was 56.4. Seven days and 3 months postoperatively, the mean EQ-VAS scores improved to 65.8 and 72.5, respectively (*p* < 0.05).

The preoperative neurological examination revealed neurological deficits in 12 patients. Seven patients presented with ankle dorsiflexion weakness, whereas plantar flexion weakness was observed in two patients. Mixed neurological symptoms involving both motor weakness and sensory disturbances were present in three patients. Prior to surgery, three patients reported limited walking distance due to neurogenic claudication.

No postoperative neurological deterioration was observed. Improvement in neurological status was noted in eight patients. Postoperative genitofemoral nerve neuropraxia, which represents one of the most frequently reported neurological complications associated with LLIF procedures, was not observed in the present study.

The mean operative time was 45 min (range: 30–70 min). The mean intraoperative blood loss was 50 mL (range: 20–140 mL); no patient required perioperative blood transfusion. The mean length of hospital stay was 3 days (range: 2–5 days).

**Figure 4:**
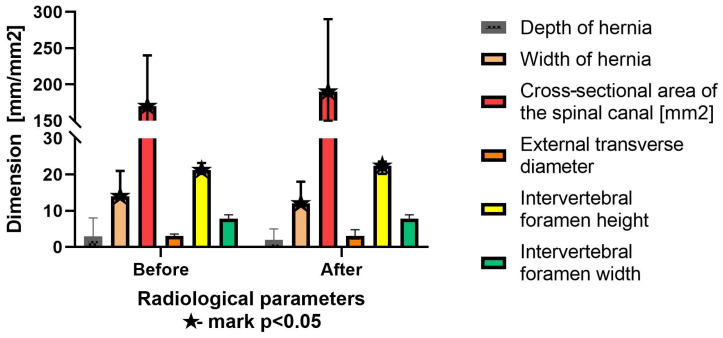
Morphological parameters on pre- and postoperative MRI scans.

Radiographic outcomes are presented in Table 2. The mean intervertebral disc height increased from 8.2 mm preoperatively to 12.2 mm postoperatively (*p* < 0.05). At 3-month follow-up, the mean disc height remained stable at 12.0 mm. The mean lumbar lordosis angle was 41.5° preoperatively, 44.5° one day after surgery, and 43.5° at 3 months postoperatively. The mean segmental lordosis improved from 11.4° preoperatively to 14.8° one day after surgery (*p* < 0.05) and was maintained at 13.1° at the 3-month follow-up (*p* < 0.05). Table 3 presents the preoperative MRI measurements, including spinal canal cross-sectional area, disc herniation depth and width, and intervertebral foraminal dimensions. The corresponding postoperative MRI findings are shown in Table 4 and Figure 4.

The most common complication was vertebral endplate injury with subsequent cage subsidence into the vertebral body, which was observed in two patients (5%). Implant migration was identified on follow-up radiographs in two patients (5%); in both cases, supplemental posterior transpedicular fixation was subsequently performed. No wound healing complications were observed. Skin sutures were removed between postoperative days 14 and 17. No injuries to neural structures, major vessels, ureter, abdominal organs, or the dura mater were noted.

## 4. Discussion

Numerous studies have confirmed the effectiveness of indirect lumbar decompression and interbody fusion performed using a lateral approach. This technique is particularly useful in the treatment of degenerative and discogenic spinal disorders associated with pain, neurogenic claudication, and neurological deficits. The application of minimally invasive lateral approaches to the lumbar spine reduces the risk of injury to paraspinal muscles and ligamentous structures, decreases postoperative pain, and facilitates wound healing and postoperative rehabilitation [1,2,5,17].

The effectiveness of indirect decompression does not appear to depend solely on the severity of spinal stenosis. Fujibayashi et al. demonstrated that patients with more severe preoperative stenosis achieved greater radiographic improvement on postoperative MRI scans [18]. Oliveira et al. reported significant improvements in all radiographic parameters following LLIF, including a mean increase in intervertebral disc height of 41.9% [19]. Similar observations have been reported in studies evaluating spinal canal decompression on MRI, demonstrating a mean increase in spinal canal cross-sectional area of approximately 33.1% following surgery [20].

Elowitz et al. demonstrated a correlation between the restoration of the intervertebral disc height and indirect decompression of the spinal canal. In their study, the anteroposterior diameter of the spinal canal increased by an average of 54%, whereas the cross-sectional area increased by approximately 83 mm^2^ (143%) [21]. Similarly, Shigeto et al. reported a significant increase in disc height at the L2–L3, L3–L4, and L4–L5 levels in 53 patients treated with LIF. These authors additionally observed a reduction in posterior disc bulging and ligamentum flavum thickness, resulting in an enlargement of the spinal canal area [22].

Tessitore et al. demonstrated an average increase in intervertebral disc height of 55% in a cohort of 20 patients, as well as improvement in the segmental spinal alignment [20]. Likewise, Hijikata et al. reported favourable effects of indirect decompression on both sagittal and coronal balance. In their study, the mean disc height increased from 6.0 mm to 10.4 mm, which correlated with an improvement in the segmental Cobb angle and a reduction in spinal deformity related to vertebral rotation [23].

Most authors have confirmed the high effectiveness of indirect decompression in the treatment of foraminal stenosis. In a retrospective study, Alimi et al. observed an average increase in foraminal height of 3.1 mm (20%) following LIF [6]. Isaacs et al. demonstrated an increase in foraminal height of 2.16 mm on the operative side and 1.39 mm on the contralateral side in patients with moderate stenosis. In cases of severe lumbar stenosis (Schizas type D), the foraminal height increased by 2.11 ± 1.75 mm (12.20 ± 10.28%) on the approach side and by 2.05 ± 1.83 mm (11.83 ± 10.82%) on the contralateral side [24].

Similarly, Tessitore et al. demonstrated asymmetry in foraminal enlargement depending on the surgical approach, which is likely related to annulus fibrosus release during the procedure. The mean foraminal area increased from 115.7 mm^2^ preoperatively to 136.5 mm^2^ postoperatively [20].

It is noteworthy that radiographic evidence of implant osseointegration has been consistently reported in the majority of published studies. The assessment of osseointegration is one of the most important parameters for evaluating treatments success, as it is associated with improved functional outcomes and a reduced rate of complications. The absence of pain and spinal stability are the primary clinical indicators of successful implant integration. CT provides the most accurate assessment of implant osseointegration, as it allows for the visualization of bridging bone formation as well as bone resorption at the bone–implant interface. MRI plays a complementary role in the assessment of osseointegration; it enables the detection of fluid or soft tissue at the bone–implant interface, bone marrow edema, and inflammatory response. Such findings on MRI are suggestive of micromotion at the bone–implant interface and provide indirect evidence of incomplete osseointegration [20,21,22,25,26,27].

In the present study, follow-up MRI demonstrated significant enlargement of the spinal canal area and reductions in disc protrusion width and depth. However, no significant changes were observed in the width of the lateral recesses or foramina (Figure 2). Interbody fusion significantly influenced sagittal alignment parameters. Postoperative radiographs obtained immediately after surgery demonstrated the restoration of the disc height as well as improvements in both local and segmental lordosis. However, follow-up radiographs obtained 3 months postoperatively demonstrated a partial loss of segmental lordosis correction, accompanied by a slight reduction in disc height and an attenuation of local lordotic correction.

Several studies have identified factors that may negatively affect clinical and radiographic outcomes following LLIF procedures. Cage subsidence related to poor bone quality or vertebral endplate injury is among the most commonly reported complications. The secondary narrowing of the spinal canal due to hypertrophy of the ligamentum flavum and progressive degeneration of posterior spinal elements has also been described. The reported incidence ranges from 10 to 30%, depending on the definition used and the radiographic criteria applied for assessment. The highest risk has been reported in patients with a low bone mineral density and those with an intraoperative endplate injury. Equally important is the appropriate selection of implant size and its positioning within the intervertebral disc space. This complication results in a significant reduction in intervertebral height, leading to decreases in segmental lordosis and foraminal height. No negative effect on the rate of osseointegration was observed. Implant migration was reported less frequently, with an incidence ranging from 0.5 to 3%. Cases requiring revision surgery to reposition the implant are rare in the literature. The most common causes of failed osseointegration include the use of an undersized implant, injury to the intraoperative ligamentous structures, and spinal instability [21,28]. In the present study, implant instability with migration was observed in two patients, while cage subsidence into the adjacent vertebral bodies occurred in another two patients. In these cases, delayed posterior spinal stabilization was performed, resulting in pain reduction and facilitating implant osseointegration. The necessity of supplemental posterior fixation following lateral interbody fusion remains controversial. In contrast to the opinion presented by Emami et al., we believe that additional posterior stabilization may not be necessary in selected cases involving single-level degenerative pathology. In cases of postoperative instability or implant migration, supplemental posterior fixation may be performed as a secondary procedure. Several authors have emphasized that appropriately designed large-footprint interbody cages provide sufficient disc space distraction, segmental stability, and the correction of both coronal and sagittal alignment [29].

In the present study, rapid postoperative functional improvement was observed. The mean ODI score decreased from 51.2 preoperatively to 14.9 at 3-month follow-up, while the mean EQ-VAS score improved from 56.4 to 72.4. Furthermore, the mean pain intensity decreased from 7.1 preoperatively to 1.8 at 3 months after surgery. Therefore, surgical treatment was associated with significant pain reduction and functional improvement within a relatively short postoperative period. Moreover, these beneficial effects increased progressively over time following surgical intervention (Table 1; *p* < 0.05).

In the present series, no neurological complications were observed. According to the published literature, the most commonly reported complications are sensory disturbances involving the anterior thigh and groin, as well as weakness of the hip flexor muscles. The overall incidence of neurological complications has been reported to range from 10 to 30%. The incidence of neurological complications is influenced by the level of surgery due to psoas muscle anatomy and lumbar plexus localization. The risk of neurological injury increases at progressively lower lumbar levels and is the highest at the L4–L5 level. Injury to the genitofemoral nerve is the most commonly reported neurological complication following surgery to the lumbar spine using the lateral approach. Lamartina et al. suggested that the retraction of the iliopsoas muscle should not exceed 30 min to reduce the risk of lumbar plexus neuropraxia [30]. The visualized separation of the iliopsoas muscle fibres within the anatomically safer anterior portion of the muscle, followed by posterior retraction, may potentially reduce the risk of neural injury. The short follow-up period and the lack of a control group preclude drawing conclusions regarding the long-term stability and safety of the methods used. The beneficial impact of the modified surgical approach requires further investigation in larger patient cohorts, and the findings of the present study should be considered preliminary.

### Limitations of This Study

The present study has several important limitations. First, its retrospective design is associated with an inherent risk of selection and observational biases. Second, the study group was relatively small, which can be explained by the limited number of patients undergoing single-level, stand-alone lateral lumbar interbody fusion procedures at our institution. Additionally, the absence of a control group limits the interpretation of the findings and precludes direct comparison with alternative approaches. Finally, the relatively short follow-up period precludes the assessment of long-term fusion rates, implant stability, the occurrence of adjacent segment disease, late implant subsidence, and late clinical outcomes.

## 5. Conclusions

Lumbar discectomy and interbody fusion performed through the modified lateral retroperitoneal approach provides good short-term postoperative outcomes and appears to be a safe and effective surgical technique. LLIF results in the enlargement of the spinal canal area, restoration of intervertebral disc height, and indirect decompression of the neural foramina.

Stand-alone, single-level LLIF may provide satisfactory clinical and radiographic outcomes in carefully selected patients with degenerative lumbar disease. Appropriate patient selection and preservation of endplate integrity appear critical for minimizing subsidence and revision risk.

In this study, the most common complication was early implant migration requiring secondary posterior transpedicular stabilization.

In this study, no neurological complications were observed, particularly injury to the genitofemoral nerve. This finding may be attributable to the technique used for preparing the lateral aspect of the intervertebral disc; however, further studies involving larger patient cohorts are required to confirm this observation.

## Figures and Tables

**Table 1:** Functional outcomes after surgical treatment for lumbar spine discopathy with LLIF.

	Before Surgery	7 Days After Surgery	3 Months After Surgery
VAS	7	3 *	1.5 *
ODI	51.2	31.8 *	14.9 *
EQ-VAS	56.4	65.8 *	72.4 *

Values are presented as means. * A *p*-value < 0.05 is considered significant.

**Table 2:** Radiographic parameters before and after surgical treatment for lumbar spine discopathy with LLIF.

	Before Surgery	1 Day After Surgery	3 Months After Surgery
ADH	8.2 mm	12.2 mm *	12 mm
LLA	41.5°	44.5° *	43.5°
LSA	11.4°	14.8° *	13.1° *

Values are presented as means, * A *p*-value < 0.05 is considered significant.

**Table 3:** Parameters on the preoperative MRI scans of the spine.

	L1–L2	L2–L3	L3–L4	L4–L5
Depth of the hernia (mm)	2–4 (mean 3)	2–4 (mean 3)	2–5 (mean 3)	3–8 (mean 6)
Width of the hernia (mm)	11–15 (mean 12)	11–17 (mean 14)	12–20 (mean 15)	12–21 (mean 15)
Size of the spinal canal area (mm^2^)	210–130 (mean 65)	230–126 (mean 160)	240–140 (mean 180)	140–270 (mean 185)
Width of the right lateral recess (mm)	2.2–4.7 (mean 3.2)	2.4–4.7 (mean 3.2)	2.1–3.4 (mean 2.8)	2.4–3.6 (mean 2.9)
Width of the left lateral recess (mm)	2.2–4.8 (mean 3.2)	2.4–4.7 (mean 3.2)	2.2–3.4 (mean 2.8)	2.4–3.6 (mean 2.9)
Height of the right intervertebral foramen (mm)	22.3–23.2 (mean 22.8)	22.2–23.4 (mean 22.8)	20.1–21.6 (mean 21.2)	20.2–21.4 (mean 20.8)
Height of the left intervertebral foramen (mm)	22.3–23.2 (mean 22.8)	22.2–23.6 (mean 22.8)	20.2–21.6 (mean 21.2)	20.2–21.4 (mean 20.8)
Width of the right intervertebral foramen (mm)	7.2–8.6 (mean 7.6)	7.1–8.2 (mean 7.4)	6.6–8.5 (mean 8.1)	6.8–8.9 (mean 7.4)
Width of the left intervertebral foramen (mm)	7.2–8.8 (mean 7.6)	7.1–8.2 (mean 7.4)	6.4–8.5 (mean 8.0)	6.6–8.9 (mean 7.3)

**Table 4:** Parameters on the postoperative MRI scans of the spine.

	L1–L2	L2–L3	L3–L4	L4–L5
Depth of the hernia (mm)	1–3 (mean 2)	1–3 (mean 2)	1–3 (mean 2)	2–5 (mean 3)
Width of the hernia (mm)	10–15 (mean 11)	10–15 Mean (11)	10–17 (mean 13)	11–18 (mean 14)
Size of the spinal canal area (mm^2^)	220–160 (mean 180)	240–170 (mean 185)	250–180 (mean 210)	150–290 (mean 220)
Width of the right lateral recess (mm)	2.2–4.8 (mean 3.2)	2.4–4.7 (mean 3.2)	2.1–3.4 (mean 2.8)	2.4–3.6 (mean 2.9)
Width of the left lateral recess (mm)	2.2–4.7 (mean 3.2)	2.4–4.7 (mean 3.2)	2.2–3.4 (mean 2.8)	2.4–3.6 (mean 2.9)
Height of the right intervertebral foramen (mm)	22.3–23.6 (mean 23.1)	22.2–23.6 (mean 23.1)	20.1–22.8 (mean 22.4)	20.2–22.4 (mean 22.0)
Height of the left intervertebral foramen (mm)	22.3–23.6 (mean 23.0)	22.2–23.6 (mean 23.0)	20.2–22.6 (mean 22.1)	20.2–22.2 (mean 21.9)
Width of the right intervertebral foramen (mm)	7.2–8.6 (mean 7.6)	7.1–8.2 (mean 7.4)	6.6–8.5 (mean 8.1)	6.8–8.9 (mean 7.4)
Width of the left intervertebral foramen (mm)	7.2–8.8 (mean 7.6)	7.1–8.2 (mean 7.4)	6.4–8.5 (mean 8.0)	6.6–8.9 (mean 7.3)

Values are presented as means. A *p*-value < 0.05 is considered significant compared with the values presented in Table 3.

## Data Availability

All data generated and/or analyzed during this study are included in this manuscript.

## Abbreviations

LLIF	Lateral lumbar interbody fusion
ODI	Oswestry Disability Index
VAS	Visual analogue scale
EQ-VAS	Euro-Quality of Life Visual Analog Scale
MRI	Magnetic resonance imaging
CT	Computer tomography
STIR	Short tau inversion recovery
